# Detection of Pancreatic Ductal Adenocarcinoma-Associated Proteins in Serum

**DOI:** 10.1016/j.mcpro.2023.100687

**Published:** 2023-11-27

**Authors:** T. Mamie Lih, Liwei Cao, Parham Minoo, Gilbert S. Omenn, Ralph H. Hruban, Daniel W. Chan, Oliver F. Bathe, Hui Zhang

**Affiliations:** 1 Department of Pathology, Johns Hopkins University School of Medicine, Baltimore, Maryland, USA; 2 Department of Pathology, Cumming School of Medicine, University of Calgary, Calgary, Alberta, Canada; 3 Departments of Computational Medicine & Bioinformatics, Internal Medicine, Human Genetics, and School of Public Health, University of Michigan, Ann Arbor, Michigan, USA; 4 The Sidney Kimmel Comprehensive Cancer Center, Johns Hopkins University, Baltimore, Maryland, USA; 5 The Sol Goldman Pancreatic Cancer Research Center, Johns Hopkins University, Baltimore, Maryland, USA; 6 Department of Oncology, Johns Hopkins University School of Medicine, Baltimore, Maryland, USA; 7 Department of Urology, Johns Hopkins University School of Medicine, Baltimore, Maryland, USA; 8 Departments of Surgery and Oncology, Cumming School of Medicine, University of Calgary, Calgary, Alberta, Canada

**Keywords:** pancreatic cancer, serum, mass spectrometry, glycoproteomics, data-independent acquisition

## Abstract

Pancreatic ductal adenocarcinoma (PDAC) is one of the most lethal cancer types, partly because it is frequently identified at an advanced stage, when surgery is no longer feasible. Therefore, early detection using minimally invasive methods such as blood tests may improve outcomes. However, studies to discover molecular signatures for the early detection of PDAC using blood tests have only been marginally successful. In the current study, a quantitative glycoproteomic approach *via* data-independent acquisition mass spectrometry was utilized to detect glycoproteins in 29 patient-matched PDAC tissues and sera. A total of 892 N-linked glycopeptides originating from 141 glycoproteins had PDAC-associated changes beyond normal variation. We further evaluated the specificity of these serum-detectable glycoproteins by comparing their abundance in 53 independent PDAC patient sera and 65 cancer-free controls. The PDAC tissue-associated glycoproteins we have identified represent an inventory of serum-detectable PDAC-associated glycoproteins as candidate biomarkers that can be potentially used for the detection of PDAC using blood tests.

## Highlights

• Global and glycoproteomic analyses are applied to PDAC sera and matched tissues.
• 173 proteins show differential expression in both tissues and sera.
• An inventory of PDAC-associated candidates can be used for PDAC detection.
• Tissue-based serum analysis is essential for detecting disease proteins in serum.

## Graphical Abstract

**Figure:**
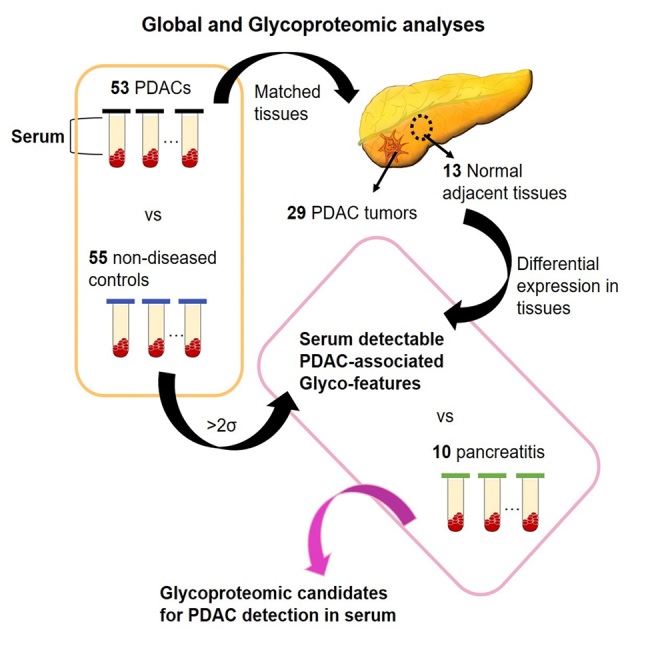

## In Brief

Proteomic and glycoproteomic characterizations of sera from pancreatic ductal adenocarcinoma (PDAC) patients and matched pancreatic cancer tissues reveal serum detectable PDAC-associated proteins and glycoproteins. However, all serum detectable protein changes are subset of the changes detected by glycoproteomics, indicating that glycoproteomics allows connectivity studies for tissue-released glycoproteins in sera. This study provides a list of glycoproteins for PDAC detection in tissues and sera. The novel tissue-targeted serum glycoproteomic approach connects the changes in disease sites to the molecular alterations in patient sera.

Pancreatic ductal adenocarcinoma (PDAC) is one of the most lethal cancer types. In 2022, there were an estimated 62,210 new cases and 49,830 deaths in the United States (1). It is predicted that PDAC will be the second leading cause of cancer death, after lung cancers, by 2030 (2, 3). PDACs are clinically aggressive, and most PDACs respond poorly to chemotherapy or immunotherapy, contributing to their high lethality. Moreover, PDAC is often detected at an advanced stage, when potentially curative resection is no longer feasible (4). Approximately 80 to 85% of the patients are diagnosed with unresectable cancers, which are either locally advanced or have metastasized distantly, leading to a low overall 5-year survival rate less than 11% (4, 5, 6). Early detection of PDAC, when surgery is still feasible, may improve these outcomes.

Although numerous efforts have been made, there are no validated, reliable methods for the early detection of PDAC (5). Imaging tests, such as multiphase computerized tomography, endoscopic ultrasound, and endoscopic retrograde cholangiopancreatography, are used clinically in symptomatic patients but are not appropriate for screening the general population (5, 6, 7). Carbohydrate antigen 19.9 is a serum tumor marker clinically used to monitor the progression of PDAC, but it is not universally elevated in PDAC patients, and it is often elevated in patients with other diseases (*e.g.*, pancreatitis and biliary obstruction). Therefore, it is not a reliable marker for the early detection of PDAC (5).

Recent advances in mass spectrometry (MS) technology allow large-scale, high-throughput proteomic characterization of tissues from various cancer types, including PDAC, revealing an inventory of proteins expressed in tumor tissues (6, 8, 9, 10, 11, 12). Blood tests are appealing since they can be used to detect, minimally invasively, proteins secreted or shed from tumors.

Previous studies have recognized the somatic genetic alterations that drive PDAC progression, especially activating point mutations in *KRAS*, as well as molecular heterogeneity of tumors based on multi-omics data (6, 13). To improve the early detection of PDAC, in this study, we utilized quantitative glycoproteomic and proteomic approaches *via* data-independent acquisition (DIA) MS to conduct paired detection of PDAC proteins in sera from 53 patients with PDAC from whom we had 29 cases with matched tumor tissues and 13 case-matched normal adjacent pancreatic tissues (NATs). By comparing glycopeptides (*i.e*., deglycosylated N-linked glycopeptides) and glycoproteins from the serum sample of each of the PDAC patients to 55 nondiseased controls, we discovered 892 glycopeptides derived from 141 glycoproteins that had PDAC-associated changes beyond two standard deviations away from the mean of nondiseased controls in serum and were highly expressed in PDAC serum samples with both matched tumor tissue and case-matched NAT. Glyco-signatures that can be detected in both tissue and serum indicate the potential clinical utilities of these glyco-features in PDAC detection using blood tests.

## Experimental Procedures

### Experimental Design and Statistical Rationale

The main objective of the current study is to investigate the correlation between tissue and matched serum from the same patients with PDAC. Statistically significant results of glycopeptides/glycoproteins that are detected across all patients may not show practical significance in the real clinical setting since the molecular basis of a disease can vary among patients or patient subgroups. We evaluated the heterogeneity among the individuals with PDAC (n = 53) by comparing their serum samples to disease-free controls (n = 55). Glycopeptides and glycoproteins exhibiting abundance beyond the normal distribution (*i.e.*, more than 2 standard deviations away from the mean of the nondiseased control group) in serum from each PDAC patient were identified as potential PDAC-associated signatures. To investigate if these potential PDAC-associated signatures found in the serum samples reflect features evident in tumor tissues, we further examined the serum samples along with matched tumor tissues (n = 29, including 13 with case-matched NATs). Additionally, a comparison was made between these signatures and the glycopeptides/glycoproteins detected in serum samples from patients with pancreatitis (n = 10) to ensure these signatures are highly associated with PDAC.

### Serum Specimens

The specimens were from the University of Calgary GI/HPB Tumor Bank, a study approved by the Health Research Ethics Board of Alberta (Ethics HREBA.CC-16–0769). Informed consent was obtained from all participants. Sera were collected in gold top vacutainers (BD Biosciences), which contained a clot activator and a gel for serum separation. Samples were spun down within 6 h of collection and frozen at −20 °C until shipping to Johns Hopkins University for MS analysis. Sera were collected immediately prior to the patients undergoing the surgery for a pancreatic mass. Additional details of sample collection can be found in our previous work (14). A total of 118 human serum samples (53 from patients with PDAC, 55 from nondiseased controls, and 10 from patients with pancreatitis) were shipped to Johns Hopkins University for this study. The 55 nondiseased controls were selected and matched to the pancreatic cancer patients based on age and gender. All subjects were de-identified before sending the samples for proteomic and glycoproteomic analyses.

### Digestion of Serum Proteins

Serum (16.6 ul) was mixed with 109 ul of lysis buffer (8 M urea, 75 mM NaCl, 50 mM Tris (pH 8.0), 1 mM EDTA, 2 μg/ml aprotinin, 10 μg/ml leupeptin, 1 mM PMSF, 1:100 (vol/vol) Phosphatase Inhibitor Cocktail 2, 1:100 (vol/vol) Phosphatase Inhibitor Cocktail 3, 10 mM NaF, and 20 μM PUGNAc). Serum proteins were reduced with 5 mM dithiothreitol at 37 °C for 1 h and then alkylated with 10 mM iodoacetamide at 25 °C for 45 min in the dark. To reduce the urea concentration to 2 M, the reaction buffer was diluted by 4-fold with 50 mM Tris HCl pH 8.0. The proteins were digested with Lys-C (FUJIFILM Wako Chemicals U.S.A. Corporation) in an enzyme to substrate ratio of 1:50 at 25 °C for 2 h and were then treated with trypsin (Promega) in an enzyme to substrate ratio of 1:50 at 25 °C for 14 h. Formic acid (50%) was added to the solution to adjust pH (final pH < 3).

### C18 Desalting

The peptides were desalted with C18 solid-phase extraction cartridge (100 mg sorbent per cartridge, Waters tC18 SepPak). The cartridge was conditioned by acetonitrile (ACN), 50% ACN (0.1% FA), and 0.1% TFA. The peptides were loaded twice to the conditioned C18 Cartridge. The cartridge was washed with 4 × 1 ml of 0.1% TFA. The peptides were then eluted with 600 ul of 50% ACN (0.1% FA), and 5% of the eluted peptides were saved for global proteomic analysis.

### Glycopeptide Capture

The glycopeptides were enriched using the SPEG method (15). In brief, the remaining 95% eluted peptides were oxidized with sodium periodate (10 mM) in dark at room temperature for 1 h with gentle shaking. The peptides were cleaned up with C18 solid-phase extraction as described above. Hydrazide resin (90 ul) was washed with 3 × 1 ml water and then mixed with the peptides. One percent of aniline was then added to the solution. The reaction was incubated at room temperature for 1 h with gentle shaking. The resin was washed with 3 × 1 ml of 50% ACN, 1.5 M NaCl, water, and 25 mM NH_4_HCO_3_ by vortexing for 20 s. The resin was then resuspended in 200 μl 25 mM NH_4_HCO_3_. Three microliters of PNGase F were added to the solution to release glycopeptides from the resin. The supernatant was collected. The resin was washed with 3 × 100 μl of 50% ACN by shaking vigorously for 1 min, and the supernatant was collected and combined with the previously collected supernatant, dried, and cleaned by C18 desalting system as described above.

### LC-MS/MS

Unlabeled tryptic peptides and glycopeptides were spiked with index retention time peptides (Biognosys) and subjected to DIA analysis. Approximately 1 ug of peptides was separated on an in-house packed 28 cm x 75 mm diameter C18 column (1.9 mm Reprosil-Pur C18-AQ beads (Dr Maisch GmbH); Picofrit 10 mm opening (New Objective)) lined up with an Easy nLC 1200 UHPLC system (Thermo Fisher Scientific). The column was heated to 50 °C using a column heater (Phoenix-ST). The flow rate was set at 200 nl/min. Buffer A and B were 3% ACN (0.1% FA) and 90% ACN (0.1% FA), respectively. The peptides were separated from 0% B to 30% B gradient in 121 min. Peptides were eluted from the column and nanosprayed directly into Orbitrap Fusion Lumos mass spectrometer (Thermo Fisher Scientific). The mass spectrometer was operated in a data-independent mode. The DIA segment consisted of one MS1 scan followed by 30 MS2 scans (*i.e.*, 30 windows with overlaps). Additional parameters were as follows: MS1: m/z range – 350 to 1650, Resolution – 120K, RF Lens – 30%, AGC Target 1.0e6, Max IT – 60 ms, Cycle time – 4.62; MS2: m/z range – 300 to 1600, Resolution – 30K, AGC Target – 1.0e6, Max IT – 120 ms, Cycle time – 4.62.

### Glycoproteome and Global Proteome Data Processing

The DIA raw files of serum samples were searched against a UniProt/SwissProt human protein database (v2019, 20,417 entries) *via* the direct DIA approach in Spectronaut (version 15.6, Biognosys) to identify and quantify glycopeptides and global proteins. Mass tolerance of MS and MS/MS was set as dynamic with a correction factor of one. Source specific index retention time calibration with a local (nonlinear) RT regression was applied. Cross run normalization was disabled and the precursors were filtered by a Q value cutoff of 0.01 (which corresponds to an FDR of 1%). Carbamidomethyl (C) was set as fixed modification. Acetyl (Protein N-term) and oxidation (M) were set as variable modifications. Additional variable modification, deamidation (N), was added for the glycopeptide search since we used PNGase F-treated glycopeptides in this study. The quantity of a peptide was a sum of the quantity of its top three precursors, whereas the quantity for a precursor was calculated by summing the area of its top three fragment ions at MS/MS level. Detailed Spectronaut setting and identification can be found in the supplemental File S1 and supplemental Tables S1 and S2.

### Data Analysis

The expression matrices of serum samples (glycopeptides and global proteins) exported from Spectronaut (abundance >100) were log-transformed and median normalized. Batch correction was applied to glycoproteomic data and global proteomic data of serum *via* ComBat (v3.36) (16). To detect the variations among the patients with PDAC, the abundances of glycopeptides/proteins were compared to the nondiseased controls by first computing the standard deviation for each glycopeptide/protein in the normal control samples followed by filtering out the glycopeptide/proteins with abundance less than or equal to two standard deviations away from the mean of the nondiseased control group for each patient with PDAC. The same filtering procedure was applied to the pancreatitis serum samples. The global proteomic expression matrix of tissues was from our previous publication (6), and fold change of each protein was calculated between a tumor and its own paired NAT. For each serum marker panel (either composed of one candidate marker or multiple candidate markers), its discriminatory power through logistic regression was evaluated using receiver operating characteristic (ROC) analysis. The candidate marker data (missing values were median imputed) were z-transformed prior to ROC analysis. We used bootstrap resampling (n = 500) of the data to construct and evaluate the predictive model of a serum marker panel to ensure statistical stability of the results. The mean ROC curves were depicted based on bootstrap resampling results, and an area under the curve (AUC) was computed for the mean ROC curve. All the analyses were carried out in R (version 3.5). The predictive models were built using caret (version 6.0–85) (17), and ROC curves were generated using pROC (version 1.13) (18). The glycosylation occupancy changes for the serum-detectable PDAC-associated glycopeptides (from glycoproteomic data) and the corresponding protein levels (global proteomic data) were obtained by calculating the median log2 fold change between 53 serum samples from patients with PDAC and 55 serum samples from nondiseased controls and performing Wilcoxon rank sum test with *p*-values adjusted using Benjamini-Hochberg method.

## Results

### Detection of Glycopeptides for Each PDAC Serum Sample

Efforts to identify serum biomarkers are often directed at markers that are uniformly present across all samples. As a result, many previous serum proteomic or glycoproteomic studies have disregarded proteins or peptides identified in a very few samples (6, 19). We have taken a different approach. Since patients and their cancers are heterogeneous (20), we have considered features that may only be present in some individuals. To this end, in this study, a glycopeptide or a protein detected in at least one of the sera from patients with PDAC was considered as a part of a larger signature that, when summed together, may account for the individual variations in PDAC sera and facilitate broad detection of PDAC.

**Fig. 1:**
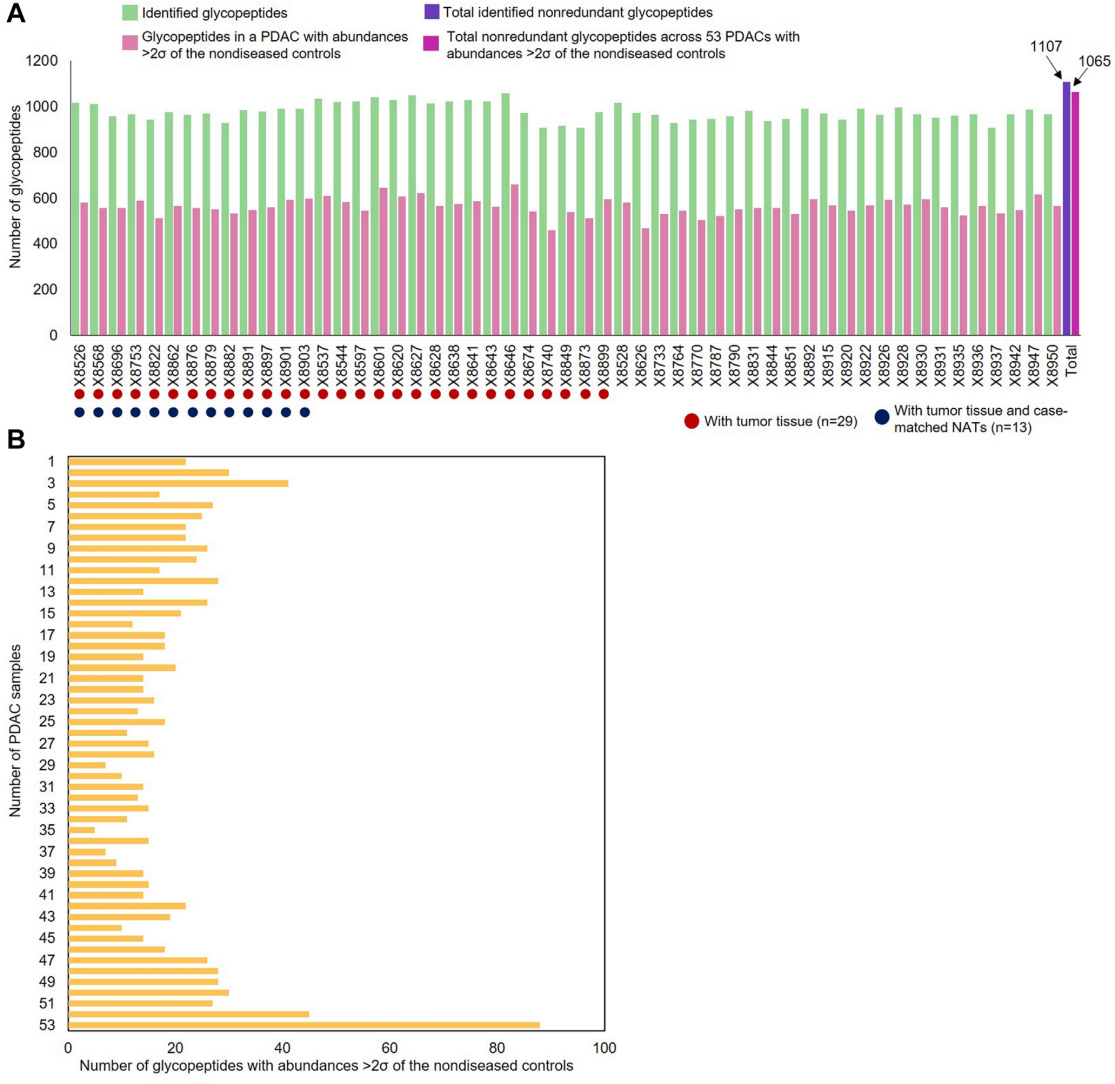
**Detection of glycopeptides from each PDAC serum**. *A*, the number of glycopeptides detected in each of the 53 PDAC sera and the number of altered glycopeptides compared to the glycopeptides from nondiseased control serum group (abundance >2σ of nondiseased controls). *B*, the numbers of altered glycopeptides detected in at least 1, 2, 3, … and all 53 of PDAC sera. Glycopeptides refer to deglycosylated N-linked glycopeptides. NATs, normal adjacent tissues; PDAC, pancreatic ductal adenocarcinoma.

A total of 1107 nonredundant glycopeptides (corresponding to 308 glycoproteins) were identified across the 53 PDAC sera, 55 sera of nondiseased controls, and 10 pancreatitis sera (supplemental Table S3). Of these nonredundant glycopeptides, a mean of 978 glycopeptides was quantified in each PDAC serum sample (Fig. 1*A*). To identify glycopeptides that were most likely PDAC-related, the abundances of glycopeptides in the serum samples from patients with PDAC were compared to the abundances in the nondiseased controls, and only those glycopeptides with an abundance of more than two standard deviations (>2σ) away from the mean of the nondiseased control group were selected (Fig. 1*A*). A distribution of the numbers of glycopeptides found in the sera from the 53 PDAC patients is shown in Figure 1*B*. Of the nonredundant glycopeptides with abundances >2σ of the nondiseased control group, 535 glycopeptides were identified in >50% of the sera from patients with PDAC, but only 88 glycopeptides were detected in all 53 samples. In all, this analysis demonstrated a high degree of variability in the glycoproteomic profile of the serum of patients with PDAC and highlighted the potential power of including a broader array of markers beyond those uniformly overexpressed in all samples.

### Association Between Sera From Patients With PDAC and Matched Tumor Tissues

**Fig. 2:**
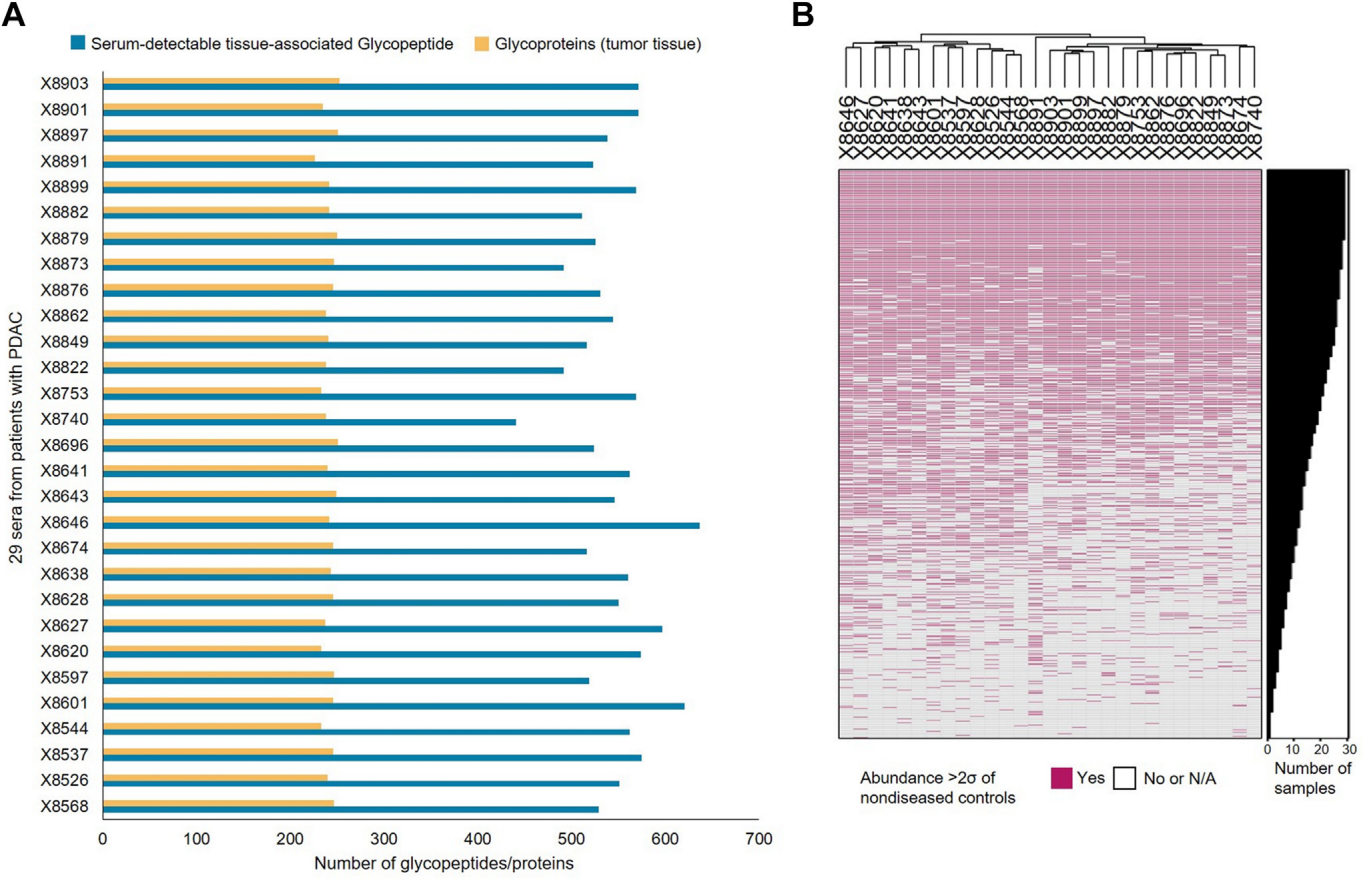
**Glycopeptide detection in PDAC tumors and patient-matched sera**. *A*, total number of serum-detectable altered glycopeptides and corresponding proteins detected in each of the 29 PDAC sera. *B*, heatmap showing the variations in the expression profiles of serum-detectable glycopeptides among the 29 PDAC sera. Unsupervised hierarchical clustering was applied to the samples (*i.e*., columns). The rows (glycopeptides) of the heatmap are in descending order according to the number of PDAC sera showing high expression of the glycopeptides. Glycopeptides refer to deglycosylated N-linked glycopeptides. PDAC, pancreatic ductal adenocarcinoma.

Matched tumor tissue from our previous study was available from 29 of the 53 patients with PDAC (Fig. 1*A*) (6). We wanted to explore whether the altered serum glycopeptides reflected features evident in patient-matched tumors. Indeed, for each individual patient, >50% of serum glycopeptides with their cognate proteins were also present in the global proteome of the PDAC tissues. Thus, these serum-detectable glycopeptides were considered as tissue associated. Figure 2*A* shows the distributions of the tissue-associated glycopeptides and corresponding proteins identified in each PDAC serum and its matched tumor tissue.

Not surprisingly given the individual variation in the PDAC tissue proteome, there was also individual variation in the corresponding serum glycopeptides in PDAC patients, shown in Figure 2*B*. Of the glycopeptides altered in sera, 45 glycopeptides originating from 37 glycoproteins were only detected in one of the 29 PDAC serum samples. Only 120 glycopeptides were identified in all 29 serum samples from patients with PDAC. These results further illustrated the variability of signatures of individual PDAC patients.

### Examining Serum-Detectable Glycoproteins That Are PDAC Associated

**Fig. 3:**
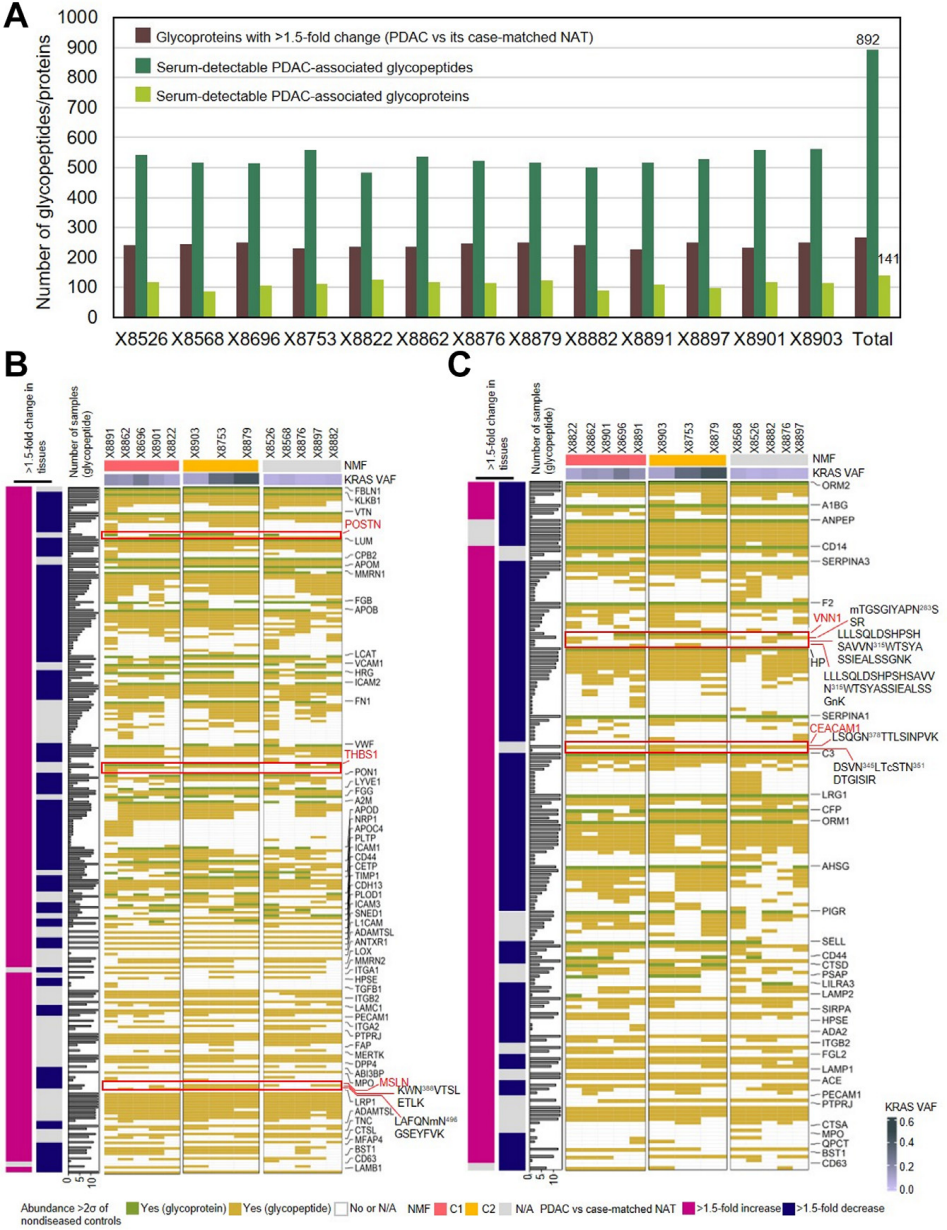
**PDAC-associated glycopeptides and proteins.***A*, distribution of PDAC-associated glycopeptides and proteins matched the glycoproteins with >1.5-fold change on the tissue level (a tumor tissue *versus* its own NAT) in each of the 13 PDAC sera with matched tumor tissues and case-matched NATs. *B*, heatmap showing proteins and glycopeptides associated with extracellular structure organization and/or cell–substrate adhesion. The samples were split according to the multiomic clusters (NMF) from our previous study. *C*, heatmap showing proteins and glycopeptides associated with neutrophil-mediated immunity. The samples were split according to the NMF clusters. Glycopeptides refer to deglycosylated N-linked glycopeptides. APOB, apolipoprotein B-100; CEACAM1, carcinoembryonic antigen-related cell adhesion molecule 1; MSLN, mesothelin; NATs, normal adjacent tissues; POSTN, periostin; PDAC, pancreatic ductal adenocarcinoma; THBS1, thrombospondin-1; VNN1, vanin-1.

We next investigated whether the glycopeptides/glycoproteins that were detected in serum were PDAC associated by examining normal adjacent pancreatic tissues. Among the 29 serum samples from patients with PDAC that had matched tumor tissues, 13 also had case-matched NATs available (Fig. 1*A*). The relative abundance of each glycoprotein was compared in PDAC tissue and case-matched NAT. Any glycoproteins with >1.5-fold change in any of the 13 PDAC tissues compared to the case-matched NATs were considered PDAC-altered glycoproteins. By filtering the serum-detectable PDAC tumor glycopeptides/glycoproteins based on the PDAC-altered glycoproteins, 892 glycopeptides from 141 glycoproteins were found in any of the 13 serum samples in patients with PDAC (Fig. 3*A*). Similar to the previous analysis, the glycopeptide/glycoprotein signature was variable between individual patients (supplemental Fig. S1).

Upon further examination of the biological processes identified *via* overrepresentation enrichment analysis in WebGestal (21), the majority of the PDAC-associated glycoproteins were involved in extracellular structure organization, cell–substrate adhesion, or immune-related processes (supplemental Table S4). Glycoproteins, such as mesothelin (MSLN), periostin (POSTN), and thrombospondin-1 (THBS1), are associated with extracellular structure organization and/or cell–substrate adhesion. In our previous work, we determined that their abundance at the protein level was elevated in PDAC tissues compared to NATs and normal ductal tissues (6, 22). Corresponding to that, we found that the abundance of POSTN and THBS1 was higher in several sera from PDAC patients, but they were not elevated in all PDAC patients (Fig. 3*B*). In contrast, glycopeptides of MSLN (KWN^388^VTSLETLK and LAFQNM[+15.99]N^496^GSEYFVK) were detected with abundance >2σ of disease-free sera in nine of the 13 PDAC sera (Fig. 3*B*). Moreover, we observed glycoproteins and glycopeptides associated with neutrophil-mediated immunity (Fig. 3*C*), such as vanin-1 (VNN1) and carcinoembryonic antigen-related cell adhesion molecule 1 (CEACAM1). In this study, glycopeptide LLLSQLDSHPSHSAVVN^315^WTSYASSIEALSSGNK of VNN1 was increased only in two PDAC sera with abundance >2σ of the nondiseased control group, whereas another glycopeptide of VNN1, M[+15.99]TGSGIYAPN^283^SSR, was found in the 11 serum samples from patients with PDAC (Fig. 3*C*). VNN1 protein was also found to be highly abundant in six PDAC sera, five of which had a high *KRAS* VAF (≥0.0956). CEACAM1, a member of the CEA family, is associated with the activation and apoptosis of neutrophils, while the inhibition of CEACAM1 may reduce tumor progression (23, 24, 25). Interestingly, CEACAM1 protein was increased in 12 of the 13 PDACs at the tissue level but not detected in the serum using global proteomics. However, two glycopeptides of CEACAM1, LSQGN^378^TTLSINPVK (n = 13 PDAC sera) and DSVN^345^LTC[+57.02]STN^351^DTGISIR (n = 1), were increased compared to the sera of nondiseased controls (Fig. 3*C*). Although the functions involving these glycopeptides are not fully understood, our observation supported the utility of glycoproteomics in finding signatures for PDAC tissues.

### Detection of PDAC-Associated Glycoproteins in Serum From Patients With PDAC Compared to Serum Samples From Patients With Pancreatitis

**Fig. 4:**
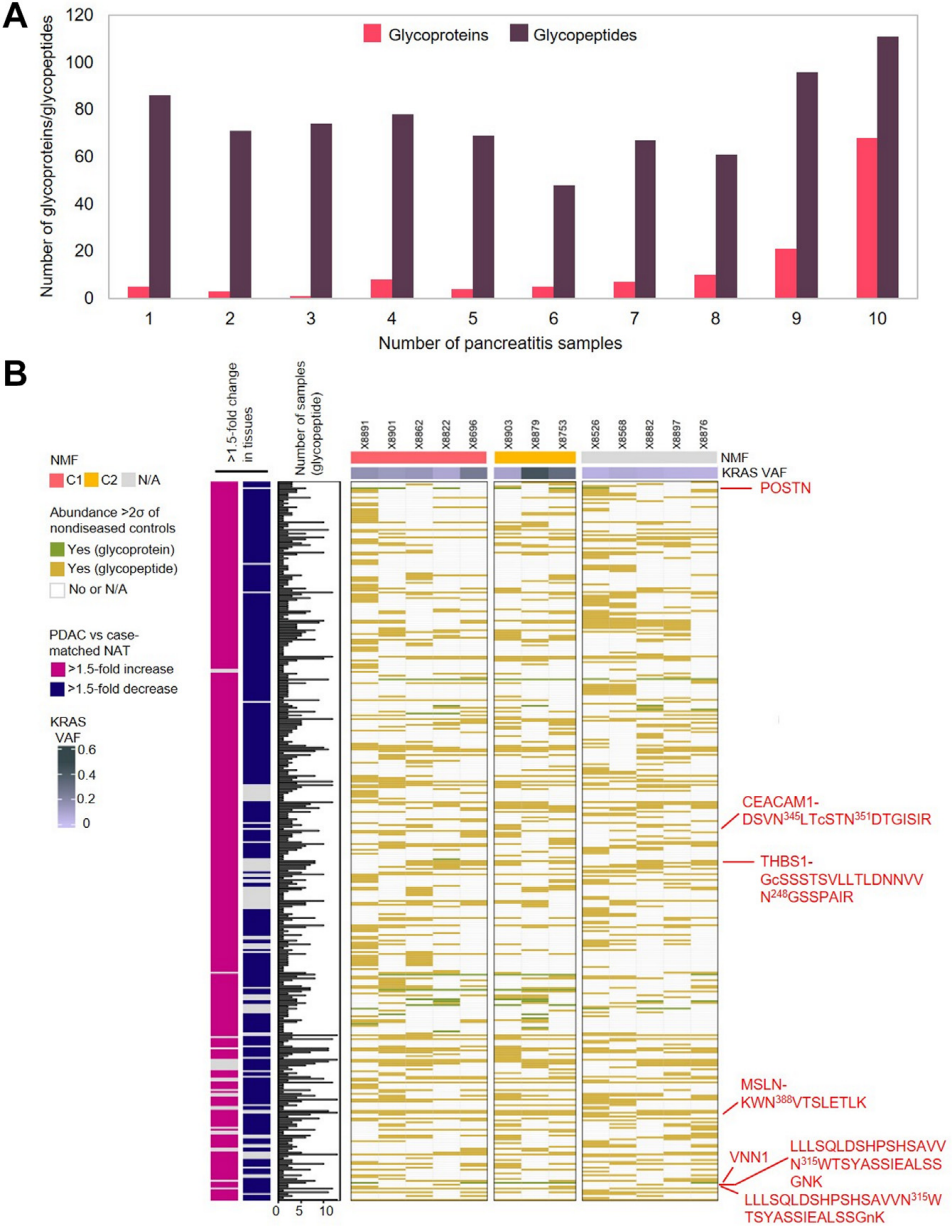
**Detectability of PDAC-associated glycopeptides and glycoproteins in 10 pancreatitis sera.***A*, the numbers of PDAC-associated glycopeptides and glycoproteins detected in at least 1, 2, …, 10 pancreatitis sera. *B*, PDAC-associated glycopeptides and glycoproteins further compared to 10 pancreatitis sera, where the samples were split according to the NMF clusters. Glycopeptides refer to deglycosylated N-linked glycopeptides. CEACAM1, carcinoembryonic antigen-related cell adhesion molecule 1; MSLN, mesothelin; NATs, normal adjacent tissues; PDAC, pancreatic ductal adenocarcinoma; POSTN, periostin; THBS1, thrombospondin-1; VNN1, vanin-1.

To investigate whether the serum-detectable PDAC-associated glycoproteins were candidate signatures for PDAC, we evaluated sera from 10 patients with pancreatitis using glycoproteomics and global proteomics. Figure 4*A* shows the distribution of the numbers of serum-detectable PDAC-associated glycopeptides and glycoproteins that could be detected in sera from patients with pancreatitis with abundance >2σ of nondiseased control group. A glycopeptide/glycoprotein was considered as a candidate signature for PDAC if it was detected in less than three pancreatitis sera. Of the 892 glycopeptides and 141 glycoproteins that were considered to originate from PDAC tumor after considering the tissue proteome, 362 glycopeptides and 18 glycoproteins could be considered as candidate signatures after comparing to the pancreatitis serum samples (Fig. 4*B* and supplemental Table S5). Aforementioned glycopeptides of CEACAM1, THBS1, MSLN, and VNN1 were also part of the serum-detectable PDAC-associated candidate signatures, further demonstrating that these glycopeptides were more associated with PDAC than they were to pancreatitis. VNN1 and POSTN were serum-detectable PDAC-associated glycoproteins. VNN1 was found in only two sera from patients with pancreatitis with abundance >2σ of nondiseased group, whereas none of the sera from patients with pancreatitis had a protein abundance of POSTN >2σ of nondiseased control group. Overall, we observed a higher percentage (∼40%) of serum-detectable glycopeptides that were PDAC associated after filtering by pancreatitis compared to glycoproteins (∼13%). The results indicated that higher sensitivity and deeper profiling in PDAC serum were better achieved using glycoproteomics than using global proteomics. Further analysis was required to determine the potential clinical utility of the serum-detectable PDAC-associated glycopeptides/glycoproteins for differentiating PDAC patients from nondiseased controls and pancreatitis patients.

### Glycopeptides and Proteins Used to Discriminate Patients With PDAC From Nondiseased Controls and Pancreatitis Patients

**Fig. 5:**
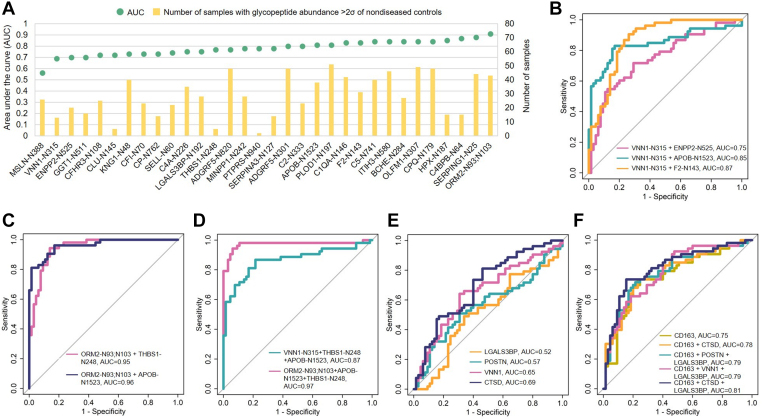
**Performance of glycopeptides and glycoproteins in distinguishing PDAC from nondiseased controls and pancreatitis**. *A*, AUC of each individual glycopeptide and total number of samples showing abundance >2σ of nondiseased controls for the particular glycopeptide. *B*, ROC curves of the panels composed of VNN1-N315 with ENPP2-N525, APOB-N1523, or F2-N143. *C*, ROC curves of the panels composed of ORM2-N93;N103 with THBS1-N248 and with APOB-N1523. *D*, ROC curves of panels composed of three glycopeptides. *E*, ROC curves of individual glycoproteins. *F*, ROC curves of CD163 and panels composed of CD163 with other glycoproteins. Additional panels composed of two or more glycopeptides can be found in supplemental Tables S7 and S8. Glycopeptides refer to deglycosylated N-linked glycopeptides. AUC, area under the curve; APOB, apolipoprotein B-100; CD163, scavenger receptor cysteine-rich type 1 protein M130; ENPP2, autotaxin; F2, prothrombin; MSLN, mesothelin; ORM2, alpha-1-acid glycoprotein 2; PDAC, pancreatic ductal adenocarcinoma; POSTN, periostin; ROC, receiver operating characteristic; THBS1, thrombospondin-1; VNN1, vanin-1.

The ability of the serum-detectable PDAC-associated glycopeptides/glycoproteins to differentiate serum samples from patients with PDAC from nondiseased controls and pancreatitis were further evaluated using ROC analysis. Since the glycopeptides and glycoproteins expressed by some PDACs may not be expressed across all PDACs, we did not require glyco-signatures used in the ROC analysis to be altered across all samples. We combined 55 sera of nondiseased controls and 10 sera from patients with pancreatitis into one group, since the sample size of pancreatitis was limited. As a solitary feature, the glycopeptide of MSLN, KWN^388^VTSLETLK, was least sensitive and specific for PDAC, with an AUC of only 0.56. Glycopeptides from VNN1 (LLLSQLDSHPSHSAVVN^315^WTSYASSIEALSSGNK, referred to as VNN1-N315 for simplicity) and THBS1 (GC[+57.02]SSSTSVLLTLDNNVVN^248^GSSPAIR, THBS1-N248) had AUC of 0.69 and 0.77, respectively (Fig. 5*A*). Moreover, glycopeptides from other proteins, such as apolipoprotein B-100 (FN^1523^SSYLQGTN[+0.98]QITGR, APOB-N1523), prothrombin (PEIN^143^STTHPGADLQENFC[+57.02]R, F2-N143), and alpha-1-acid glycoprotein 2 (QNQC[+57.02]FYN^93^SSYLNVQREN^103^GTVSR, ORM2-N93;N103), demonstrated the capability to distinguish PDAC from nondiseased controls and pancreatitis, while ORM2-N93;N103 had the highest AUC of 0.91 among all the serum-detectable PDAC-associated glycopeptides (Fig. 5*A* and supplemental Table S6).

The detection of PDAC was improved when glycopeptides were combined into two-signature panels (supplemental Table S7). For instance, the glycopeptide VNN1-N315 had an AUC of 0.69 as an individual marker. However, the performance was enhanced when VNN1-N315 was combined with ENPP2-N525 (autotaxin, PAPNN^525^GTHGSLNHLLR) (AUC=0.75), APOB-N1523 (AUC = 0.85), or F2-N143 (AUC = 0.87) (Fig. 5*B*). Moreover, when ORM2-N93;N103 was combined with glycopeptides such as THBS1-N248 or APOB-N1523, AUCs were further improved to 0.95 and 0.96 (Fig. 5*C*). Including an additional glyco-signature into panels, for example, VNN1-N315+APOB-N1523 with THBS1-N248, or ORM2-N93;N103+THBS1-N248 with APOB-N1523, could strengthen the overall performance compared to using just two signatures (Fig. 5*D* and supplemental Table S8).

The glycoproteins were evaluated individually and in different combinations as well (supplemental Table S9). Using the global protein expression of POSTN and VNN1 for ROC analysis, we observed the performance of POSTN (AUC = 0.57) and VNN1 (AUC = 0.65) on the global protein level was much lower than analyses at the glycopeptide level (Fig. 5, *A* and *E*). The performance was not improved even when combining POSTN and VNN1 into a two-signature panel. On the other hand, scavenger receptor cysteine-rich type 1 protein M130 (CD163) achieved an AUC of 0.75. Combining CD163 and cathepsin D, an AUC of 0.78 was obtained (Fig. 5*F*). By further combining CD163 and galectin-3-binding protein with POSTN, VNN1, or cathepsin D into three-signature panels, the AUCs were improved to a range from 0.79 to 0.81 (Fig. 5*F*). Nonetheless, serum marker panels composed of glycopeptides (individually and in combination) could separate serum samples from patients with PDAC from nondiseased and from patients with pancreatitis more effectively than the panels consisting of glycoproteins. Thus, the changes in glycopeptides may be more specific to PDAC compared to the overall protein expression.

### Glycosylation Occupancy Changes of PDAC-Associated Glycopeptides

The expression profile of glycopeptides may be attributable to either the expression of their corresponding proteins or the glycan occupancy of the glycosylation occurs at different glycosylation sites. Glycosylation occupancy is the percentage of the glycosylation at a specific glycosylation site. It could be calculated by the absolute quantitation of the occupied glycosite dividing the sum of absolute quantitation of the peptide from glycan occupied site and the nonoccupied peptide at the site. In this study, we did not measure the absolute quantities of the peptides at different glycosylation sites. The data only allow us to determine the glycosylation occupancy changes, which refers to the observed changes in each glycosylation site but not changes in their total protein levels. We computed the glycosylation occupancy changes for each of the 362 serum-detectable PDAC-associated glycopeptides (from glycoproteomic data) and its corresponding protein levels (global proteomic data) as described in the Experimental Procedures.

Among the 362 serum-detectable PDAC-associated glycopeptides, we observed glycosylation increases on 62 glycopeptides (adjusted *p*-value <0.05 and >1.5-fold increase) since their corresponding proteins either (a) showed no significant changes (adjusted *p*-value >0.05) between PDACs and nondiseased controls or (b) the proteins were not detected in the global proteomic data (supplemental Table S10). We selected three glycopeptides as examples to demonstrate the glycosylation occupancy changes (supplemental Fig. S2). L-selectin (SELL) has been demonstrated to promote tumor metastasis by recruiting leukocytes to locations where tumor embolization occurs (26). In our study, we found higher glycosylation in PDAC sera compared to nondiseased controls for SELL at glycosite N60 (SELL-N60, top panel of supplemental Fig. S2*A*); however, we did not observe significant global protein expression change of SELL (bottom panel of supplemental Fig. S2*A*). Moreover, SELL-N60 showed good performance (AUC > 0.7) for distinguishing PDAC from nondiseased controls and pancreatitis (Fig. 5*A*). Leukocyte immunoglobulin-like receptor subfamily A member 3 (LILRA3) is part of a family of leukocyte cell-surface receptors associated with activating or inhibiting signals (27, 28), but its role in cancer is not fully understood. The glycosite N281 of LILRA3 was elevated in the sera of PDAC patients compared to nondiseased controls in our study (top panel of supplemental Fig. S2*B*), while the protein itself was unchanged (bottom panel of supplemental Fig. S2*B*). A previous study showed high expression of poliovirus receptor (PVR) *via* immunohistochemistry staining of human pancreatic cancer tissues and its association with clinical outcome of the patients (29). In this study, we only found the glycopeptide of PVR (PVR-N120, top panel of supplemental Fig. S2*C*) but not the protein itself, regardless the sera from PDAC patients or nondiseased controls (bottom panel of supplemental Fig. S2*C*). Taken together, we observed that the glycosylation changes could be independent from the global expression of the corresponding proteins for some serum-detectable PDAC-associated glycopeptides indicating the glycosylation occupancy changes.

## Discussion

PDACs are aggressive malignancies that are most frequently diagnosed at an advanced stage. To enhance survival outcomes, one essential strategy is to devise methods for detecting PDAC at an early stage, when curative-intent surgery is still feasible. We designed this study with the main objective of investigating the potential connectivity between tissue and serum by examining whether the PDAC-associated glyco-features identified in the tumor tissue are detectable in the matched serum from the same patients. Glyco-features that could be detected in both tissue and serum indicate the potential clinical utilities of these glyco-features in PDAC detection using blood tests. In the current study, instead of the conventional statistical analysis, we focused on any glycopeptides (from glycoproteomics)/glycoproteins (from global proteomics) in the sera of PDAC patients with abundance >2σ away from the mean of the nondiseased controls. There were two main reasons for such an approach. First, we observed high tumor heterogeneity of pancreatic cancers in our previous proteogenomic study (6, 13). Second, the conventional statistical analysis could provide some evidence that serum-detectable glyco-features were different in the cancer group compared to the noncancer group but would be limited depending on the sample sizes for tumors in different subtypes. Moreover, the conventional approach usually relies on the *p* value and fold change to determine whether a feature is significant, which would neglect glycoprotein changes that do not show significance in the group comparison but may still play a biological role in one or more subtypes of pancreatic cancers. In this study, we were able to identify PDAC-associated glycopeptides and glycoproteins that, in combination, could potentially be used for detection of PDAC by using our approach. Those same features could be useful for discriminating benign and malignant pancreatic masses, which would require further investigation. Finally, we have identified patient-specific variations that may have some utility in personalizing treatment strategies.

In this study, we demonstrate the possibility of achieving a panel of PDAC-associated glyco-signatures (proteins or glycopeptides) that, in combination, can be used to indicate the likelihood that a patient has pancreatic cancer. The majority of the glyco-signatures were only highly expressed in one or few samples (Fig. 1). The variations in expression profiles of the serum-detectable PDAC tissue-associated glycopeptides suggests a high variability in the glycoproteomic profile among serum samples from patients with PDAC and the need for approaches that compensate for this variability. Many elevated glyco-signatures in the sera from patients with PDAC are involved in extracellular structure organization and/or cell–substrate adhesion, such as MSLN, POSTN, and THBS1. Abnormal glycosylation can interfere with the modeling of extracellular matrix and cell adhesion, thus enhancing tumor cell migration (30, 31). Moreover, POSTN and THBS1 are related to pancreatic cancer development and patient survival (32, 33, 34, 35). The glycopeptide of THBS1 (THBS1-N248) was identified as one of the potential markers for detection of PDAC in this particular cohort. On the other hand, while MSLN is a potential diagnostic/prognostic biomarker for PDAC which can contribute to tumor cell proliferation and invasion (36, 37), the glycopeptide of MSLN (MSLN-N388) did not demonstrate enough power to differentiate PDAC from nondiseased controls and pancreatitis.

Furthermore, recruitment of neutrophils has been observed in PDAC, associated with tumor growth and angiogenesis in the tumor microenvironment (38, 39). Among the proteins predicted to be involved in immune-related biological processes, CEACAM1 and VNN1 are two intriguing PDAC-associated glycoproteins detected in our data (tissue and serum). Studies have shown CEACAM1 as a promising serum biomarker for PDAC (23, 40). However, we found CEACAM1 upregulated in PDAC tumor tissues but not detected in PDAC sera in this particular cohort. Nevertheless, increasing glycosylation levels of the glycopeptides of CEACAM1 were observed in PDAC sera, as LSQGN^378^TTLSINPVK of CEACAM1 was highly expressed across 13 PDAC sera with matched tissues compared to the nondiseased control group; this glycopeptide could be a candidate for PDAC detection.

VNN1 has shown its involvement in inflammation and lipid metabolism as well as a potential biomarker for pancreatic cancer-associated new-onset diabetes (26, 27, 28). The glycopeptide of VNN1 (VNN1-N315) from glycoproteomic analysis could be useful for the detection of PDAC in the circulation. Combining VNN1-N315 with F2-N143 in a panel gradually increased the performance of distinguishing PDAC from nondiseased controls and pancreatitis, which has not been reported previously (Fig. 5). Moreover, ENPP2 is associated with the proliferation of PDAC cells (41), and the detection of PDAC was improved when combining the glycopeptide of ENPP2 with VNN1. Additionally, we identified several multimarker panels showing the potential for PDAC detection, including panels composed of ORM2-N93;N103+APOB-N1523. ORM2 is involved in acute inflammatory responses, a potential marker for diagnosis of prostate cancers (42). Downregulation of APOB is associated with development of hepatocellular carcinomas (43). In this study, we found the glycopeptides of APOB were abundant in patients with PDAC compared to cancer-free controls. Although the fluctuation in ORM2 and APOB may relate to acute response to the disease instead of specific markers produced by the PDAC (44, 45), adding THBS1-N248 to this particular panel could enhance the clinical utility of ORM2 and APOB in diagnosis of PDAC. Even though additional studies will be required to further determine and confirm the clinical utilities of those PDAC-associated proteins, these results suggest that detection is achievable using serum. Additionally, we used the conventional statistical approach to show the glycosylation occupancy changes of serum-detectable PDAC-associated glycopeptides, and the results have (a) demonstrated that the glycosylation changes were not influenced by the global expression of the corresponding proteins of 62 serum-detectable PDAC-associated glycopeptides indicating the glycosylation occupancy changes and (b) further supported our observation using our current analysis approach that we could achieve higher sensitivity and deeper profiling in PDAC serum using glycoproteomics than using global proteomics.

In all, our work has identified disease-associated and patient-specific glycoproteomic features that have potential utility in the diagnosis of PDAC. Further studies will be required to explore whether these features are detectable in early, low-stage disease, including in premalignant states such as pancreatic intraepithelial neoplasia and intraductal papillary mucinous neoplasms with high-grade dysplasia. Further studies on treatment response to various systemic agents as a function of the individual glycoproteomic patterns are also warranted.

## Data availability

Data are available at the Proteomic Data Commons (PDC: https://pdc.cancer.gov) and ProteomeXchange (identifier: PXD039273).

## Supplemental data

This article contains supplemental data.

## Conflict of interest

The authors declare no competing interests.
