# Supplementary material for: Detection of Pancreatic Ductal Adenocarcinoma-Associated Proteins in Serum
Source: Mol Cell Proteomics. 2023 Nov 27;23(1):100687. doi: 10.1016/j.mcpro.2023.100687 (PMC10792492; doi:10.1016/j.mcpro.2023.100687)
Supplement: Supplementary file S1 [file mmc11.pdf]

Spectronaut 15.6.211220.50606

Analysis Type: directDIA

Settings Used: BGS Factory Settings

```
├── Pulsar Search\Peptides
│   ├── Toggle N-terminal M: True
│   ├── Min Peptide Length:  7
│   ├── Max Peptide Length:  52
│   ├── Missed Cleavages:    2
│   ├── Digest Type:  Specific
│   └── Enzymes / Cleavage Rules:    Trypsin/P
├── Pulsar Search\Labeling
│   └── Channels:
│     ├── Channel 1:  False
│     ├── Channel 2:  False
│     └── Channel 3:  False
├── DIA Analysis\Data Extraction
│   ├── MS1 Mass Tolerance Strategy:       Dynamic
│   │   └── Correction Factor:       1
│   ├── MS2 Mass Tolerance Strategy:       Dynamic
│   │   └── Correction Factor:       1
│   ├── Intensity Extraction MS1:    Maximum Intensity
│   └── Intensity Extraction MS2:    Maximum Intensity
├── DIA Analysis\XIC Extraction
│   ├── XIC IM Extraction Window:    Dynamic
│   │   └── Correction Factor:       1
│   └── XIC RT Extraction Window:    Dynamic
│     └── Correction Factor:  1
├── Pulsar Search\Modifications
```

```
│   ├── Max Variable Modifications:   5
│   └── Select Modifications:
│     ├── Fixed Modifications::          Carbamidomethyl (C)
│     └── Variable Modifications: :      Acetyl (Protein N-term), Deamidation (N), Oxidation (M)
├── DIA Analysis\Calibration
│   ├── MS1 Mass Tolerance Strategy:          System Default
│   ├── MS2 Mass Tolerance Strategy:          System Default
│   ├── Precision iRT: True
│   │   ├── iRT <-> RT Regression Type:        Local (Non-Linear) Regression
│   │   └── Exclude Deamidated Peptides:      True
│   ├── MZ Extraction Strategy:         Maximum Intensity
│   └── Allow source specific iRT Calibration:  True
├── DIA Analysis\Identification
│   ├── Generate Decoys:      True
│   │   ├── Decoy Limit Strategy:       Dynamic
│   │   │   └── Library Size Fraction:   0.1
│   │   └── Decoy Method:    Mutated
│   │     └── Preferred Fragment Source:       NN Predicted Fragments
│   ├── Machine Learning:     Per Run
│   ├── Exclude Duplicate Assays:      True
│   ├── Precursor PEP Cutoff: 0.2
│   ├── Protein Qvalue Cutoff (Experiment):   0.01
│   ├── Protein Qvalue Cutoff (Run):  0.05
│   ├── Exclude Single Hit Proteins:    False
│   ├── Pvalue Estimator:      Kernel Density Estimator
│   ├── Precursor Qvalue Cutoff:        0.01
│   └── Single Hit Definition:  By Modified Sequence
├── DIA Analysis\Quantification
```

```
│   ├── Interference Correction:    True
│   │   ├── MS1 Min: 2
│   │   ├── MS2 Min: 3
│   │   ├── Exclude All Multi-Channel Interferences:    True
│   │   └── Only Identified Peptides:   True
│   ├── Protein LFQ Method: Automatic
│   ├── Major (Protein) Grouping:   by Protein Group Id
│   ├── Minor (Peptide) Grouping:   by Modified Sequence
│   ├── Minor Group Top N:  True
│   │   ├── Min:    1
│   │   └── Max:    3
│   ├── Minor Group Quantity:   Sum precursor quantity
│   ├── Major Group Top N:  True
│   │   ├── Min:    1
│   │   └── Max:    3
│   ├── Major Group Quantity:   Sum peptide quantity
│   ├── Quantity MS-Level:  MS2
│   ├── Quantity Type:  Area
│   ├── Proteotypicity Filter:  None
│   ├── Data Filtering: Qvalue
│   └── Cross Run Normalization:    False
├── DIA Analysis\PTM Workflow
│   └── PTM Localization:   False
├── DIA Analysis\Workflow
│   ├── MS2 DeMultiplexing: Automatic
│   ├── Run Limit for directDIA Library:    -1
│   ├── Method Evaluation:  False
│   ├── Profiling Strategy: None
```

```
│   └── Unify Peptide Peaks Strategy: None
├── DIA Analysis\Protein Inference
│   └── Protein Inference Workflow:   Automatic
│     └── Inference Algorithm:         IDPicker
├── DIA Analysis\Post Analysis
│   ├── Calculate Sample Correlation Matrix:  False
│   ├── Calculate Explained TIC:        None
│   ├── Differential Abundance Grouping:     Major Group (Quantification Settings)
│   │   └── Smallest Quantitative Unit:      Major Group (Quantification Settings)
│   │     └── Use All MS-Level Quantities:   True
│   ├── Differential Abundance Testing:      Un-Paired t-test
│   │   ├── Assume Equal Variance:   False
│   │   └── Group-Wise Testing Correction:   False
│   └── Run Clustering:         True
│     ├── Distance Metric:      Manhattan Distance
│     ├── Linkage Strategy:     Ward's Method
│     ├── Z-score transformation:      False
│     └── Order Runs by Clustering:    True
├── DIA Analysis\Pipeline Mode
│   ├── Post Analysis Reports:
│   │   ├── Scoring Histograms:          False
│   │   ├── Data Completeness Bar Chart:       False
│   │   ├── Run Identifications Bar Chart:     False
│   │   ├── CV Density Line Chart:       False
│   │   └── CVs Below X Bar Chart:       False
│   ├── Generate SNE File:     False
│   ├── Report Schema:         BGS Factory Report (Normal)
│   └── Reporting Unit:        Across Experiment
```

```
├── **Pulsar Search\Identification**
│   ├── Peptide FDR: 0.01
│   ├── Protein Group FDR:    0.01
│   └── PSM FDR:      0.01
├── **Pulsar Search\Tolerances**
│   └── Tolerance Parameters:
│     ├── Thermo Orbitrap:
│     │   ├── Calibration Search:        Dynamic
│     │   │   ├── MS1 Correction Factor:        1
│     │   │   └── MS2 Correction Factor:        1
│     │   └── Main Search:     Dynamic
│     │     ├── MS1 Correction Factor:          1
│     │     └── MS2 Correction Factor:          1
│     ├── TOF:
│     │   ├── Calibration Search:        Dynamic
│     │   │   ├── MS1 Correction Factor:        1
│     │   │   └── MS2 Correction Factor:        1
│     │   └── Main Search:     Dynamic
│     │     ├── MS1 Correction Factor:          1
│     │     └── MS2 Correction Factor:          1
│     └── Thermo IonTrap:
│       ├── Calibration Search:          Dynamic
│       │   ├── MS1 Correction Factor:          1
│       │   └── MS2 Correction Factor:          1
│       └── Main Search:        Dynamic
│         ├── MS1 Correction Factor:  1
│         └── MS2 Correction Factor:  1
├── **Pulsar Search\Workflow**
```

│ ├── Use DNN Predicted Ion Mobility: Auto

│ ├── Fragment Ion Selection Strategy: Intensity Based

│ └── In-Silico Generate Missing Channels: False

└── **Pulsar Search\Result Filters**

├── Precursors:

│ ├── Best N Fragments per Peptide: True

│ │ ├── Min: 3

│ │ └── Max: 6

│ ├── Channel Count: False

│ ├── Modifications: None

│ ├── Amino Acids: False

│ ├── Best N Peptides per Protein Group: False

│ ├── FASTA Matched: False

│ ├── Missed Cleavage: False

│ ├── Peptide Charge: False

│ └── Proteotypicity: False

└── Fragment Ions:

├── m/z : True

│ ├── Min: 300

│ └── Max: 1800

├── Ion Charge: False

├── Ion Loss Type: False

├── Ion Type: False

├── Ion AA Length: True

│ └── N: 3

└── Relative Intensity: True

└── Min: 5
