# Supplementary material for: Detection of Pancreatic Ductal Adenocarcinoma-Associated Proteins in Serum
Source: Mol Cell Proteomics. 2023 Nov 27;23(1):100687. doi: 10.1016/j.mcpro.2023.100687 (PMC10792492; doi:10.1016/j.mcpro.2023.100687)
Supplement: Supplementary Figures [file mmc12.pdf]

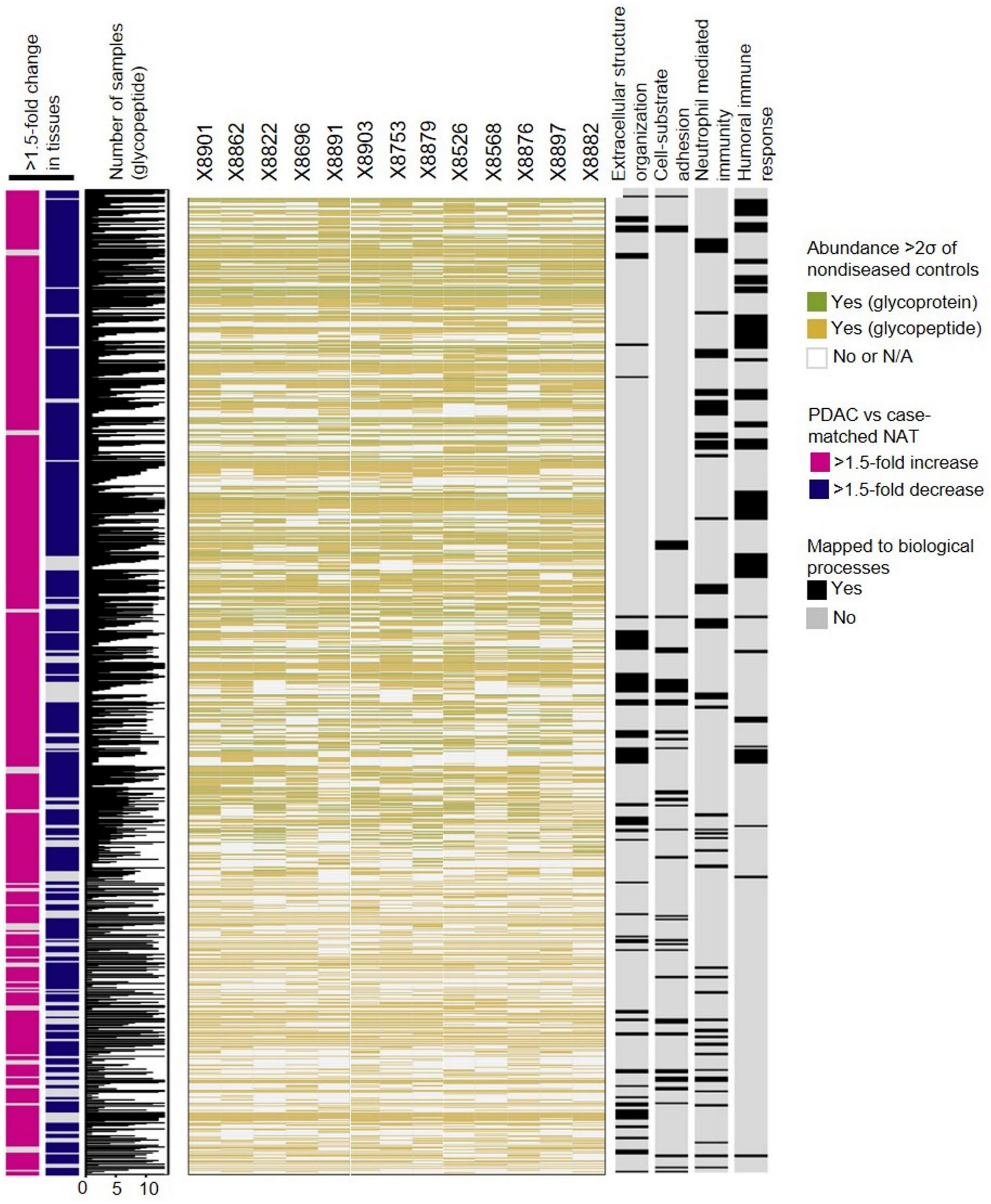

>1.5-fold change in tissues
Number of samples (glycopeptide)
0
5
10
X8901
X8862
X8822
X8696
X8891
X8903
X8753
X8879
X8526
X8568
X8876
X8897
X8882
Extracellular structure organization
Cell-substrate adhesion
Neutrophil mediated immunity
Humoral immune response
Abundance >2σ of nondiseased controls
Yes (glycoprotein)
Yes (glycopeptide)
No or N/A
PDAC vs case-matched NAT
>1.5-fold increase
>1.5-fold decrease
Mapped to biological processes
Yes
No

Figure S1. All the PDAC-associated glycopeptides and glycoproteins found in PDAC sera with matched tumor tissues and case-matched NATs. Known biological processes for each of these glycopeptides/glycoproteins are indicated. This is related to Figure 3. Glycopeptides refer to de-glycosylated N-linked glycopeptides.

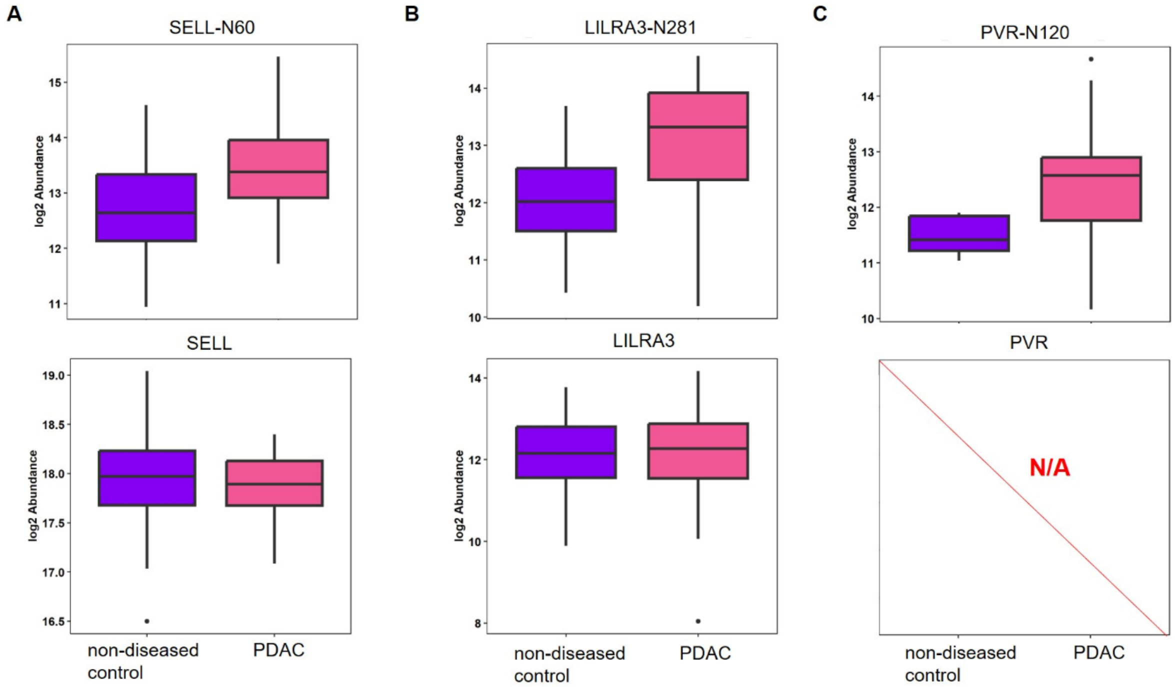

Figure S2. Glycosylation occupancy changes or glycopeptide change of identified candidate glycopeptides associated with PDAC patients. **A.** Glycopeptide occupancy change for L-selectin (SELL) at glycosite N60 (SELL-N60, top panel) and the consistent expression of the corresponding protein (bottom panel). **B.** Glycopeptide occupancy change for leukocyte immunoglobulin-like receptor subfamily A member 3 (LILRA3) at glycosite N281 (LILRA3-N281, top panel) and the consistent expression of the corresponding protein (bottom panel). **C.** Glycopeptide change for poliovirus receptor (PVR) at glycosite N120 (PVR-N120, top panel) and the corresponding protein was not detected in both PDAC and nondiseased controls (bottom panel). Glycopeptides refer to de-glycosylated N-linked glycopeptides.
